# CDK1 plays an important role in the maintenance of pluripotency and genomic stability in human pluripotent stem cells

**DOI:** 10.1038/cddis.2014.464

**Published:** 2014-11-06

**Authors:** I Neganova, K Tilgner, A Buskin, I Paraskevopoulou, S P Atkinson, D Peberdy, J F Passos, M Lako

**Affiliations:** 1 Institute of Genetic Medicine, Newcastle University, International Centre for Life, Newcastle upon Tyne NE1 3BZ, UK; 2 Centre for Integrated Systems Biology of Ageing and Nutrition, Institute for Ageing and Health, Newcastle University, Newcastle upon Tyne NE4 5PL, UK

## Abstract

Human embryonic stem cells (hESC) and induced pluripotent stem cells (hiPSC) are characterised by an unusual and tightly regulated cell cycle that has been shown to be important for the maintenance of a pluripotent phenotype. Cyclin-dependant kinase 1 (CDK1) is a key player in cell cycle regulation and particularly mitosis; however, its role has not been studied previously in hESC and hiPSC. To investigate the impacts of *CDK1* downregulation, we performed RNA interference studies which in addition to expected mitotic deficiencies revealed a large range of additional phenotypes related to maintenance of pluripotency, ability to repair double strand breaks (DSBs) and commitment to apoptosis. Downregulation of *CDK1* led to the loss of typical pluripotent stem cell morphology, downregulation of pluripotency markers and upregulation of a large number of differentiation markers. In addition, human pluripotent stem cells with reduced *CDK1* expression accumulated a higher number of DSBs were unable to activate CHK2 expression and could not maintain G2/M arrest upon exposure to ionising radiation. *CDK1* downregulation led to the accumulation of cells with abnormal numbers of mitotic organelles, multiple chromosomal abnormalities and polyploidy. Furthermore, such cells demonstrated an inability to execute apoptosis under normal culture conditions, despite a significant increase in the expression of active PARP1, resulting in tolerance and very likely further propagation of genomic instabilities and ensuing of differentiation process. On the contrary, apoptosis but not differentiation, was the preferred route for such cells when they were subjected to ionising radiation. Together these data suggest that CDK1 regulates multiple events in human pluripotent stem cells ranging from regulation of mitosis, G2/M checkpoint maintenance, execution of apoptosis, maintenance of pluripotency and genomic stability.

The high proliferation ability and maintenance of pluripotency in human pluripotent stem cells is directly linked to the regulation of core cell cycle factors.^1, 2, 3, 4, 5, 6, 7^ Human embryonic stem cell (hESCs) are derived from the inner cell mass of pre-implantation blastocysts, whereas induced pluripotent stem cells (hiPSCs) are generated from the reprogramming of somatic cells back to pluripotency. Both cell types have the potential to generate almost any cell type of the human adult organism and for this reason, it is assumed that the requirement for genomic stability is critical; however, chromosomal instabilities are often observed in hESCs and hiPSCs.^8, 9, 10^

With the aim to better understand mitotic progression and its regulation in human pluripotent stem cells, we focused on a key cell cycle regulator, cyclindependent kinase 1 (Cdk1).^11^ Although the majority of Cdks and cyclins have been shown to be largely dispensable, Cdk1 has emerged as the master regulator of mammalian cell cycle, whose role *in vivo* cannot be compensated by other closely related Cdks, including Cdk2.^12, 13, 14^ Studies performed in primary cultures and established cell lines do not always match up to the wide range of Cdk and Cyclin compensatory mechanisms observed *in vivo* and this has led to the idea that certain cell types (especially cells emerging during embryonic development) may have not developed the full spectrum of compensatory mechanisms.^15, 16^

Cdk1 is highly expressed in murine ESCs and interacts directly with Oct4, enhancing its binding to the trophoectoderm marker *Cdx2* and promoting its repression.^17^ Knockdown of *Cdk1* relieves this repression, resulting in the activation of *Cdx2* and differentiation of mouse ESCs into trophoectodermal lineages.^17, 18^ Conditional knock-out of *Cdk1* in mouse results in the arrest of embryonic development around the blastocyst stage and DNA re-replication, because of an increase in Cdk2/Cyclin A activity.^14^ Similarly, the inhibition of Cdk1 *via* a chemical inhibitor (R03306) leads to abortive endoreduplication and apoptosis in murine ESCs,^19^ suggesting an important role for this kinase in mitotic progression in ESCs. Till now, insights on CDK1 function in hESC and hiPSC are missing, despite CDK1/2 emerging as the central kinome controlling self-renewal and differentiation of these cells.^20^ The high proliferative nature of hESC and hiPSC would suggest a high dependence of these cells on CDK1 for the proper regulation of mitosis and successful completion of cell cycle. However, how CDK1 regulates and/or impacts other processes, for example, apoptosis, maintenance of pluripotency and genomic stability is unknown and forms the main focus of this manuscript.

## Results

### Downregulation of CDK1 causes pluripotent stem cell accumulation in G2 phase, loss of pluripotency and induction of differentiation

**Figure 1:**
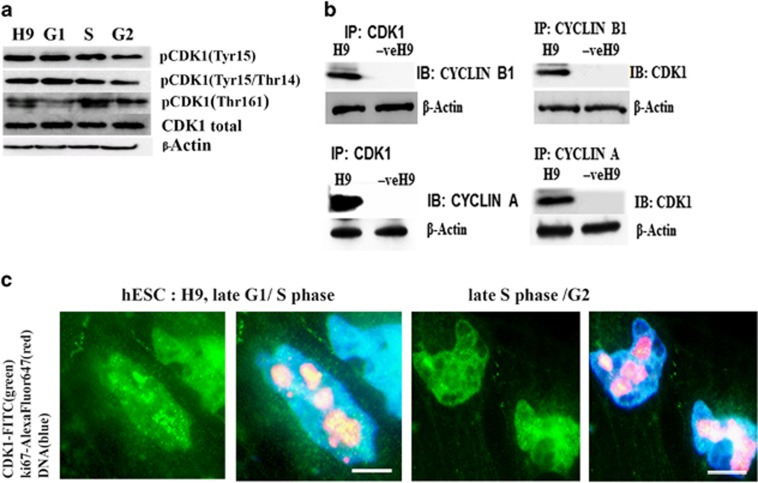
Expression and cellular localization of CDK1 in hESC. (**a**) Western blot of a total hESC lysates probed with antibodies against total CDK1 and specific phospho-residues important for cell cycle progression. Cells were synchronized at G1 (70%), S (75%) and G2 (68%) phases of the cell cycle as described before at Neganova *et al.*;^2^ H9-unsynchronized population. (**b**) Analysis of specific complex formation between CDK1/Cyclin B1 and CDK1/Cyclin A *via* immunoprecipitation in unsynchronized hESC. (**c**) CDK1 cellular localisation in H9 hESC during transition from late G1 to S phase and late S/G2 phase transition revealed by indirect immunofluorescence with anti-CDK1 antibody (green) and anti-Ki67 (red). Scale bar=10 *μ*m. Presented images are representative of at least three independent experiments

Our western blot analysis of hESC synchronised at different stages of the cell cycle shows that although total CDK1 expression does not vary through the cell cycle, the expression of the phosphorylated form (Thr161), which is required for the activation of the CDK1-CYCLIN B1 complex, is highest in S and G2 phases of the cell cycle (Figure 1a). In contrast, the expression of the Tyr15/Thr14 phosphorylated form, which results in CDK1 inactivation, is lower in S and G2 (Figure 1a). Together, these data suggest highest expression of active CDK1 in S and G2 phases of hESC cell cycle. Immunoprecipitation analysis indicates that CDK1 forms complexes with CYCLIN B1 and CYCLIN A which are key players in the regulation of mitosis (Figure 1b). Immunofluorescence analysis (with Ki67 and CDK1 antibodies) to distinguish the specific stages of the cell cycle (performed as in Becker *et al.*^21^) indicated a high nuclear CDK1 accumulation and a low cytoplasmic expression pattern (Figure 1c) during late G1/S and S-phase/G2 transition in hESC.

**Figure 2:**
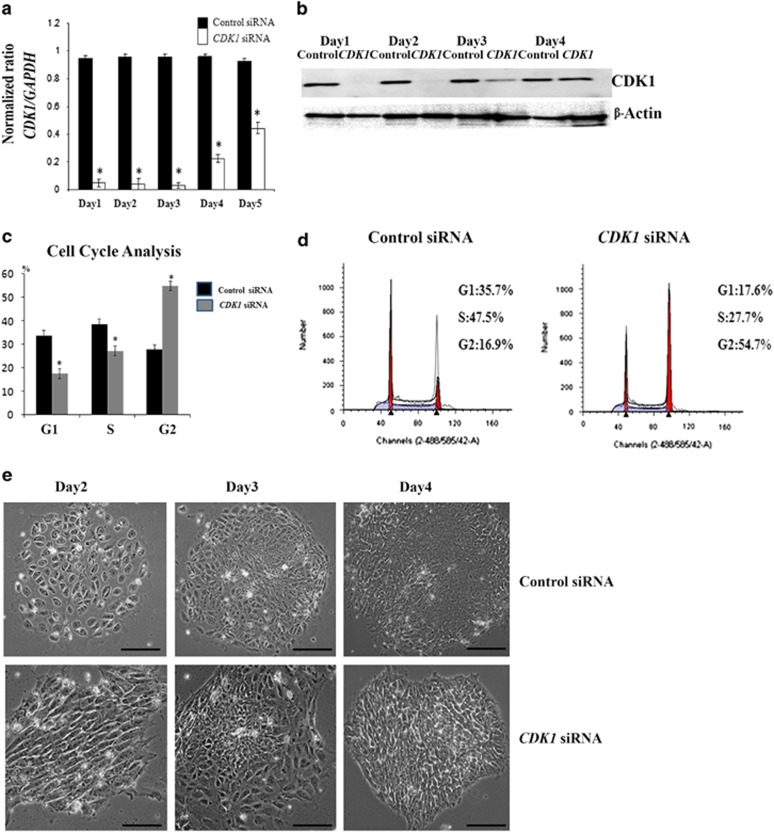
Downregulation of *CDK1* by RNAi effectively abrogates CDK1 expression and induces the accumulation of hESC at the G2 phase of the cell cycle. (**a**) Quantitative RT-PCR analysis for expression of *CDK1* from 24 to 120 h post transfection. Results are presented as average±S.E.M. (*n*=3). *T*-test analysis was carried out to assess differences in gene expression between the control and *CDK1* siRNA group, *P<0.05. (**b**) Western blot analysis of CDK1 expression. *β* –Actin was used as the loading control. The data shown are representative of six independent western blots carried out in H9 hESC line. (**c**) Graphical representation of the ModFit analysis of cell cycle distribution in the control and *CDK1* siRNA group, 48 h post transfection with siRNAs. Results are presented as average±S.E.M. (*n*=6). *T*-test analysis was carried out to assess the differences in gene expression between the control and *CDK1* siRNA group, *P<0.05. (**d**) Flow cytometric histograms showing cell cycle distribution in control and *CDK1* siRNA groups. The percentage of cells in each stage of the cell cycle is indicated in the top right corner of flow histogram. Data are representative of at least six independent experiments. (**e**) Phase-contrast images of hESC transfected with control and *CDK1* siRNAs (upper and lower panel respectively) at 2, 3 and 4 days post transfection. Scale bar=25 *μ*m

To further investigate the role of CDK1 in hESC and hiPSC, we performed RNA interference studies using small interfering RNAs (siRNAs). Combined quantitative reverse transcription-polymerase chain reaction (qRT-PCR) analysis (Figure 2a) and western blotting (Figure 2b, Supplementary Figure 1A) indicated effective *CDK1* downregulation as early as day 1 post transfection of *CDK1*-siRNAs. Immunocytochemical analysis revealed the complete loss of cytoplasmic CDK1 expression and a very significant reduction of nuclear CDK1 expression in *CDK1* siRNA-transfected cells (Supplementary Figure 2), when compared with control cells. Furthermore, we observed changes in the location of CYCLIN B1 expression (Supplementary Figure 3). We noticed that CYCLIN B1 in hESC is localized at the nuclei of control cells (Supplementary Figure 3); however, in the *CDK1* siRNA group, CYCLIN B1 was observed both at cytoplasm and the nuclei, suggesting a requirement for CDK1 activity in nuclear translocation of CYCLIN B1 in pluripotent stem cells similarly to observations made in human somatic cells.^22, 23^

**Figure 3:**
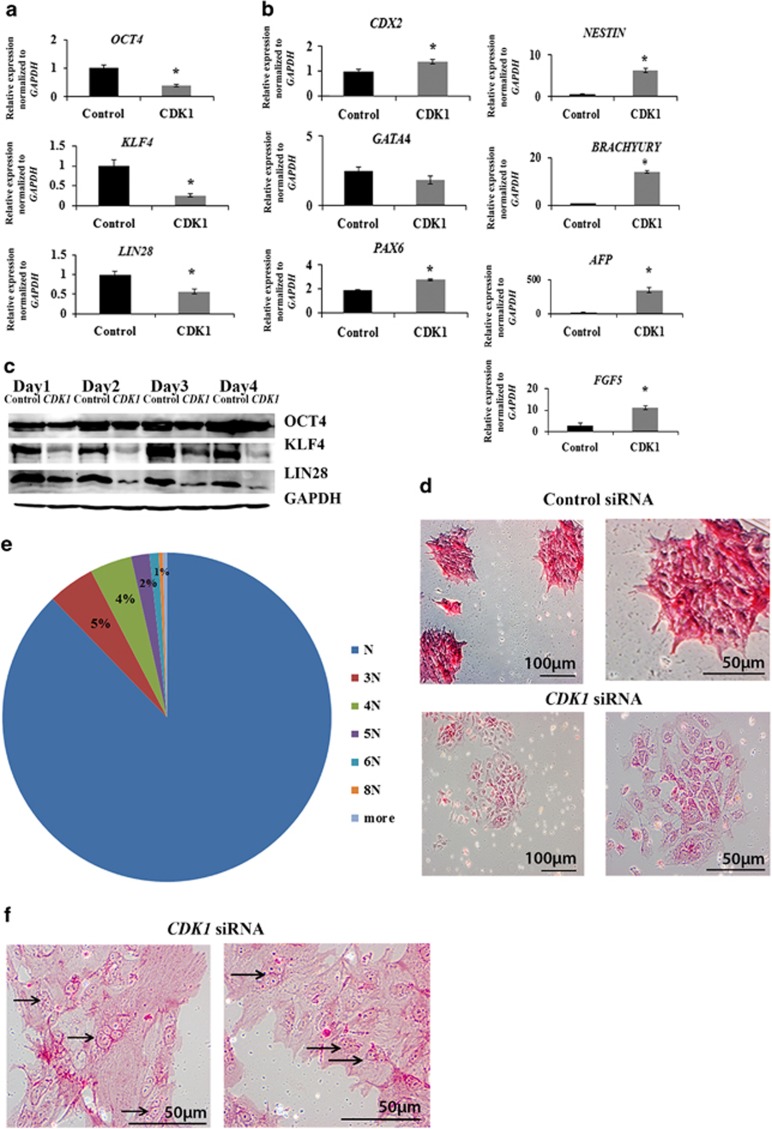
Downregulation of CDK1 leads to the loss of pluripotency, differentiation and polyploidy. (**a**) Expression analysis of main pluripotency genes by quantitative RT-PCR. Results are presented as average±S.E.M. (*n*=3). The value for the hESC transfected with control siRNA was set to 1 and all other values were calculated with respect to that. *T*-test analysis was carried out to assess the differences in gene expression between the control and *CDK1* siRNA group, *P<0.05. (**b**) Upregulation of differentiation markers analysed by quantitative RT-PCR. Results are presented as mean±S.E.M. (*n*=3). The value for the hESC transfected with control siRNA was set to 1 and all other values were calculated with respect to that. *T*-test analysis was carried out to assess the differences in gene expression between the control and *CDK1* siRNA group, *P<0.05. (**c**) Western blot analysis for the expression of key pluripotency markers. Note that the protein level of KLF4 and LIN28 was not restored by day 4 post transfection. GAPDH was used as a loading control. Data are representative of at least three independent experiments. (**d**) Alkaline-phosphatase-positive staining was observed in hESC transfected with control siRNA (upper row) but differentiated morphology and lack of typical staining was observed in cells transfected with *CDK1* siRNAs (lower panel), 48 h post transfection. Images are representative of at least three independent experiments. (**e**) Representative histogram showing the percentage of polyploid cells in the *CDK1* siRNA group at 48 h post transfection. At least 300 cells were analysed in each experiment. (**f**) Examples of appearance of multinucleated cells (black arrows) on a second day after *CDK1*siRNA treatment. Scale bar=50 *μ*m

*CDK1* downregulation resulted also in cell cycle changes with hESC accumulation in G2 phase at the expense of G1 and S phases (Figures 2c and d). Similar experiments performed in hiPSC indicated the accumulation of cells in G2 phase at the expense of S phase; however, no changes in G1 phase were observed highlighting some differences between hESC and hiPSC (Supplementary Figure 1B,C). Despite this, we observed the loss of typical pluripotent stem cell morphology upon *CDK1* downregulation in both cell types (Figure 2e, Supplementary Figure 1D). Expression analysis indicated a reduction in expression of three pluripotency markers, namely *OCT4, KLF4* and *LIN28* (Figures 3a and c), and upregulation of *CDX2* (trophoectodermal marker), *PAX6* and *NESTIN* (ectodermal markers), *FGF5* (primitive ectoderm marker), *BRACHYURY* (mesodermal marker) and *AFP* (endodermal marker), suggesting the loss of pluripotency upon *CDK1* knockdown (Figure 3b). This was also confirmed by alkaline phosphatase (AP) staining for almost complete loss of AP+ colonies in the *CDK1* siRNA group that was observed when compared with control siRNA-transfected group which contained on average about 80% AP+ colonies (Figure 3d, Supplementary Figure 1D). Counting of cell nuclei also showed that a considerable proportion of cells (~14%) in the *CDK1*-siRNA transfected group became polyploid (Figures 3e and f; Supplementary Figure 1D). Cytogenetic analysis revealed the presence of abnormal and multicentric chromosomes in addition to loss and gain of whole chromosomes in 100% of metaphases obtained from the *CDK1* siRNA group, whereas control cells showed normal karyotype (Supplementary Figure 4). Together, these data indicate an important role for CDK1 in cell cycle regulation, maintenance of pluripotency and genomic stability in human pluripotent stem cells.

### Downregulation of CDK1 results in accumulation of double strand breaks (DSBs) and impairment of CHK2 activation

**Figure 4:**
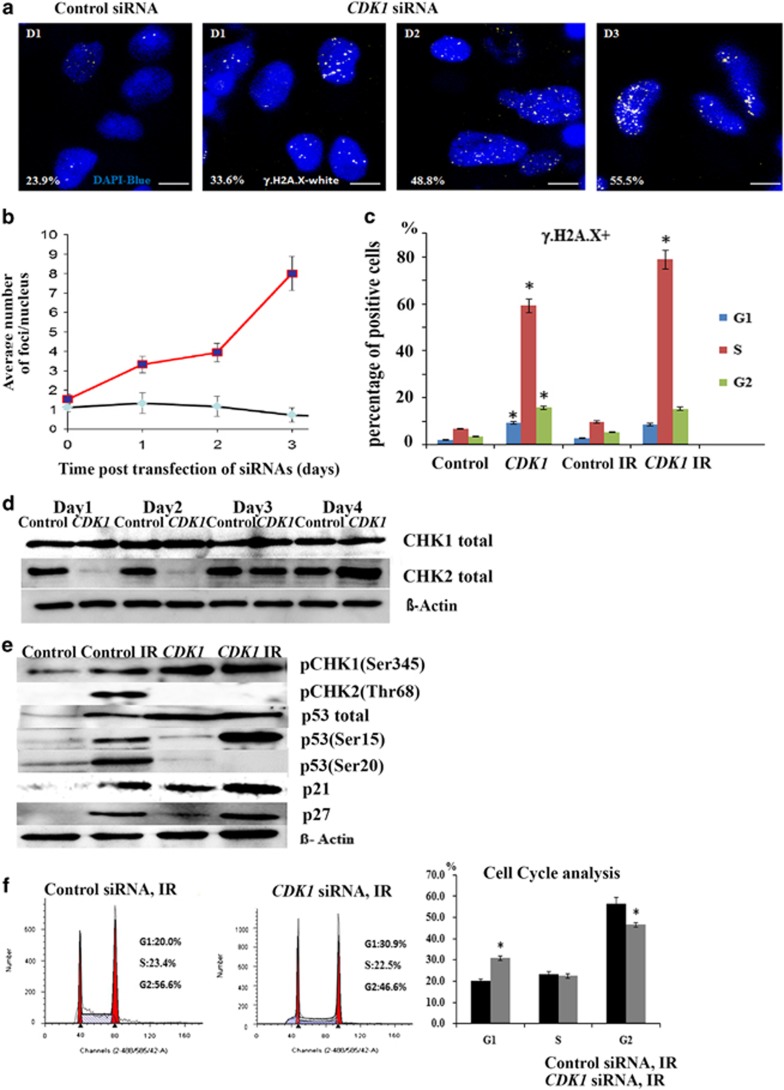
Knockdown of CDK1 induces the activation of *γ*H2.AX and downregulation of CHK2 expression in hESC. (**a**) Confocal microscopy analysis showing the presence of *γ*H2 A.X foci in hESC at 1, 2 and 3 days post transfection of control and *CDK1* siRNAs. Phosphorylated histone H2A.X (*γ* -H2A.X foci) is shown as white dots. Chromatin is stained with DAPI (blue). Scale bar=10 *μ*m, D=day. Images are representative of at least three independent experiments. Percentage of *γ*-H2A.X-positive cells is shown at the bottom. (**b**) Graphic representation of the average number of *γ*H2A.X foci per nucleus in hESC during 3-day time course post *CDK1* and control siRNAs transfections. Data areshown as average±S.E.M., *n*=3. (**c**) Flow cytometric analysis of *γ*-H2A.X on the second day post transfection and 16 h after administration of IR. Data are shown as average±S.E.M., *n*=3. (**d**) Representative images of four repeats of western blot analysis for CHK1 and CHK2 expression up to 96 h post transfection of siRNAS. *β*-Actin was used as the loading control. (**e**) Impacts of *CDK1* inhibition on the regulation of key factors involved in G2 checkpoint activation analysed by western blotting at day 2 post transfection and after 6 h post IR on the same day (shown in the figure as IR group). *β*-Actin served as the loading control. The data represent at least three independent experiments. (**f**) Representative flow cytometric histograms at 2 days post transfection+6 h post IR. The percentage of cells in each stage of cell cycle was calculated using ModFit. Graphic representation of these data is shown on the right hand panel. Results are presented as mean±S.E.M. (*n*=3). *T*-test analysis was carried out to assess the differences in gene expression between the control and *CDK1* siRNA group, *P<0.05

Under normoxic culture conditions, hESC accumulate a small number of DSBs (Figures 4a and b;^3^). However, upon *CDK1* knockdown, we observed a significant increase both in the percentage of hESC with DSBs (Figure 4a) and the number of DSB foci per cell (Figures 4a and b), corroborating data previously published in mammalian somatic cells.^24, 25^ To further confirm this, we carried out *γ*-H2A.X detection by flow cytometric analysis (Figure 4c). It is evident that upon *CDK1* downregulation, there is a significant increase in the number of accumulated DSBs (Figures 4a and c); however, this is not accompanied by increased apoptosis in the *CDK1* siRNA group when compared with the control (Figure 7d). When analysed under the context of cell cycle regulation, 59% of S-phase cells were positive for *γ*-H2A.X, in contrast to control cells which showed only 6.7% of S-phase cells with *γ*-H2A.X foci (Figure 4c). We repeated the same analysis at 16 h after administrating ionising radiation (IR) (2Gy) to hESC (Figure 4c). Although there is a slightly higher DSBs accumulation in hESC with reduced CDK1 expression under IR when compared with the same group under non-IR conditions (Figure 4c), the pattern is similar with the majority of cells with *γ*-H2A.X in the S phase of the cell cycle.

Next, we investigated whether downregulation of *CDK1* affects DNA damage response signalling. We observed a slight upregulation of CHK1 (day 1–3 post transfection, Figure 4d); however, there was a very significant downregulation of CHK2 up to 48 h post transfection of *CDK1*, suggesting an important link between CDK1 function and its ability to maintain intact CHK2 expression. This effect is also observed upon the administration of IR) which leads to a significant increase in the expression of the phosphorylated form of CHK1 (Ser 345), but not the phosphorylated form of CHK2 (Thr368, Figure 4e) following the downregulation of CDK1. p53 has been shown to be activated by both CHK2 and CHK1 in response to DNA damage; however, the specificity of CHK1 *versus* CHK2 activation can be distinguished because CHK2-dependent activation of p53 results in the phosphorylation of Ser20 of p53,^26^ whereas CHK1 activation results in the phosphorylation of Ser15 of p53.^27^ It is clear from our results (Figures 4d and e) that upon downregulation of CDK1, p53 stabilisation in response to DNA damage is achieved mostly *via* activation of CHK1 and not CHK2 because only the phosphorylated Ser15 of p53 is increased in response to DNA damage.

Studies performed in murine ESC have shown that complete absence of Chk2 leads to an inability to maintain G2 arrest after IR-induced damage.^28^ In addition, Chk2 has been reported to localise aberrantly to the centrosomes in mouse ESC and failed to translocate to the nucleus after irradiation.^29^ In contrast, hESC are able to activate ATM-CHK2-p53 checkpoint signalling resulting in G2 arrest after administration of IR of the same dose.^30^ To investigate whether this G2 arrest is maintained in hESC treated with *CDK1* siRNAs, we repeat cell cycle analysis after IR administration. This analysis showed that of hESC treated with control siRNA accumulate in G2/M, corroborating published data;^30^ however, this is compromised in the *CDK1* siRNA group where a lower percentage of cells are found in the G2/M part of the cell cycle after IR (Figure 4f). It is interesting to note that the reduction of cells in the G2/M phase in the *CDK1* siRNA group is accompanied by an increase in G1 phase which is perhaps due to increases in p21 and p27 expression in the *CDK1*-siRNA-treated groups under normal and IR conditions (Figure 4e). Unlike murine ESCs, hESCs have been shown to be capable of executing G1/S checkpoint activation in response to DNA damage.^3, 31^ Hence, a mixed G1 and G2 arrest may be the response of hESC to protect their genome under conditions (such as *CDK1* knockdown) where a full G2/M arrest cannot be guaranteed.

### An important role for CDK1 in mitosis progression

**Figure 5:**
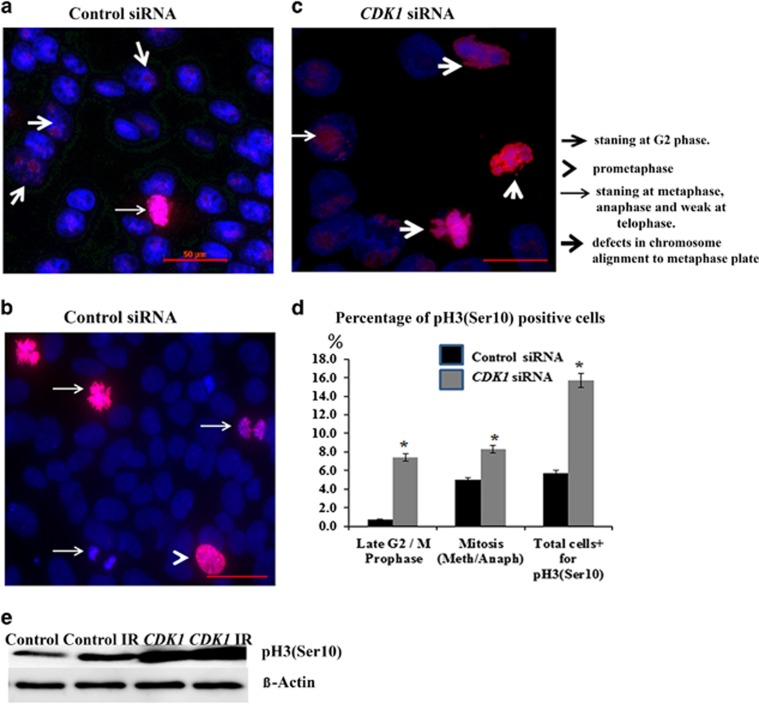
CDK1 is important for mitotic progression in hESC. (**a**–**c**) Representative images of immunofluorescence staining of mitotic marker pH3 Ser10 (red) at 2 days post transfection with control and *CDK1* siRNAs. DNA was counterstained with DAPI (blue). Scale bar=50 *μ*m; (**d**) Quantification of mitotic index calculated as percentage of cells positive for phospho-Histone 3 Ser 10 (pH3 Ser10) during mitosis progression at 2 days post transfection with control and *CDK1* siRNAs. The data represent mean±S.E.M. from three independent experiments. At least 100 cells were analysed per condition in each experiment. Statistical significance was determined by *t*- test, *P≤0.05. (**e**) Western blot analysis for pH3 Ser 10 expression at 2 days post transfection as well as 2 days post transfection+6 h post IR (shown as the IR group). *β*-Actin was used as the loading control. The data shown are representative of at least three independent western blots

Given the role of CDK1/CYCLIN B1 complex in mitosis progression, it is important to investigate whether cells with reduced levels of *CDK1* expression and correspondingly greater numbers of DSBs can pass through mitosis, as this would lead to propagation of DNA damage to daughter cells. Site-specific phosphorylation of histone H3 at Ser10 initiates during G2, peaks during metaphase and diminishes during late anaphase and early telophase.^32^ Immunofluorescence analyses (Figures 5a and c) indicated a significant increase in the number of cells expressing phospho H3 (Ser10) in the *CDK1* knockdown group (Figure 5d). These results were also corroborated by western blotting (Figure 5e). When this information was analysed in context of G2/M progression, it was evident that about half of the phospho H3+ cells from the *CDK1* knockdown group accumulated at G2/prophase and the rest in metaphase/anaphase (Figure 5d). Although accumulation at G2/ prophase would enable cells to check their DNA damage and respond, the progression to metaphase and anaphase is disconcerting and suggests that these cells can escape from proper checkpoint control and progress through mitosis whilst still carrying unrepaired DSBs.

**Figure 6:**
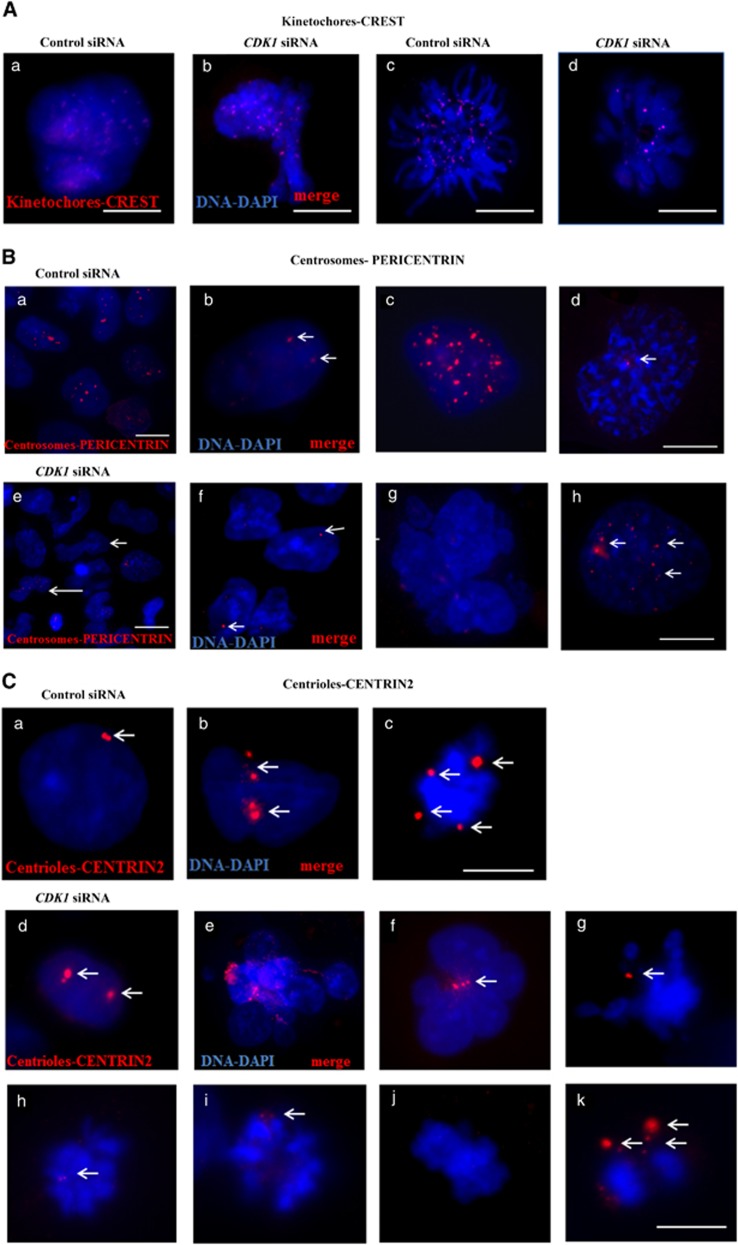
Downregulation of CDK1 leads to multiple abnormalities in kinetochore, centrosome and centriole numbers in hESC. (**A)** Specific antibody to kinetochores, CREST (red), was used for immunofluorescence staining of hESC treated with control siRNA (**a**, **c**) and *CDK1* siRNAs (**b**, **d**) at 2 days post transfection. DNA was counterstained with DAPI (blue) in all images. Examples of hESC with supernumericial kinetochores at interphase nuclei observed in control siRNA (**a**) and *CDK1* siRNA-treated cells (**b**). (**B**) Immunostaining of centrosomes with a specific antibody, Pericentrin (red). Examples of hESC with normal (**b**, **d**, **f**) and supernumerary centrosomes (**a**, **c**, **g**, **h**) at 2 days post transfection with control and *CDK1* siRNAs, respectively. Note abnormal nucleus blebbing on the *CDK1* siRNA group shown by long arrows (**e**). Centrosomes are shown by short white arrows. (**C**) Centrin2-specific antibody (red) for visualisation by immunofluorescence of centrioles at 2 days post hESC transfection with control siRNA (**a**, **b**, **c**) and *CDK1* RNAi (**d**–**k**). Normal (**a**: two centrioles; pointed by arrow) and abnormal centriole number (**b**) was observed in the control siRNA-transfected group in interphase nucleuses (**b**) and during mitosis (**c**). White arrows point to centrioles. Knockdown of *CDK1* in hESC causes abnormalities in centriole number in interphase nuclei (**d**), nuclei blebbing with abnormal centriole number (**e**, **f**) and abnormal mitoses (**g**–**k**) with wrong distribution and number of centrioles (**g**–**k**). **A**–**C**: Scale bar=5 *μ*m. Images are representative of at least three independent experiments

*CDK1* controls many aspects of mitotic chromosome behaviour, kinetochore function and spindle microtubule dynamics to ensure accurate chromosome segregation.^33, 34^ The presence of kinetochores is essential for proper chromosome segregation, because chromosome fragments that lack a kinetochore are not inherited faithfully and are quickly lost.^35^ Defects in kinetochore, in cohesion or in any of the factors that promote biorientation lead to chromosome missegregations and hence aneuploidy.^35^ We frequently observed abnormal mitosis, with chromosome missegregations, misaligned chromosomes and chromosome loss in the *CDK1* knockdown group (Figure 5c) and went on to investigate this further using immunocytochemistry with an antibody against the kinetochore-specific marker CREST. We observed proper bipolar organisation and correct numbers of kinetochores in 35.6% cells of control group; however, only 15.6% of the cells in *CDK1* siRNA group were characterized by a normal kinetochore number (for an example, see Figure 6A, panel c).

Another important event for mitosis progression is the proper function and movement of centrosomes. We performed direct visualisation of centrosomes using a PERICENTRIN-specific antibody (Figure 6B). In agreement with this previous publication, we observed normal (75% of cells in the control group and 47.3% of cells in *CDK1* siRNA group; for an example see Figure 6B, panel b) and increased numbers of centrosomes (25% of cells in control group and 52.7% of cells in the *CDK1* siRNA group (Figure 6B, panels: a, c, g, h) both in the control and in *CDK1* knockdown groups. Nevertheless, the percentage of cells with increased centrosome number in the *CDK1* siRNA group suggests an altered centrosome biogenesis upon *CDK1* downregulation, which could be due to an inability to activate CHK2 expression in response to DNA damage. In support, it has been shown that CHK2 localises at centrosomes during mitosis and alteration of CHK2 function promotes chromosome segregation errors in dividing cells, a feature which is commonly observed in cancer cells and might drive chromosomal instability and cellular transformation.^36^

Centriole function, duplication and organisation are important events for mitotic progression.^37, 38^ Immunofluorescence analysis with a centriole-specific marker, CENTRIN-2, also revealed defects in the distribution and number of this organelle upon *CDK1* downregulation (Figure 6C, panels d–k). Although normal CENTRIN-2 staining was observed in 82.7% of control cells (for an example, see Figure 6C, panel a), only 41.5% of the cells in the *CDK1* siRNA group showed normal centriole numbers. Together these data suggest that *CDK1* downregulation contributes to abnormal kinetochore, centrosome and centriole biogenesis and that all these events could be the causative factor leading to the observed chromosomal abnormalities in the *CDK1* knockdown group (Supplementary Figure 4).

### CDK1 downregulation leads to PARP1 activation but impaired apoptosis in hESC

**Figure 7:**
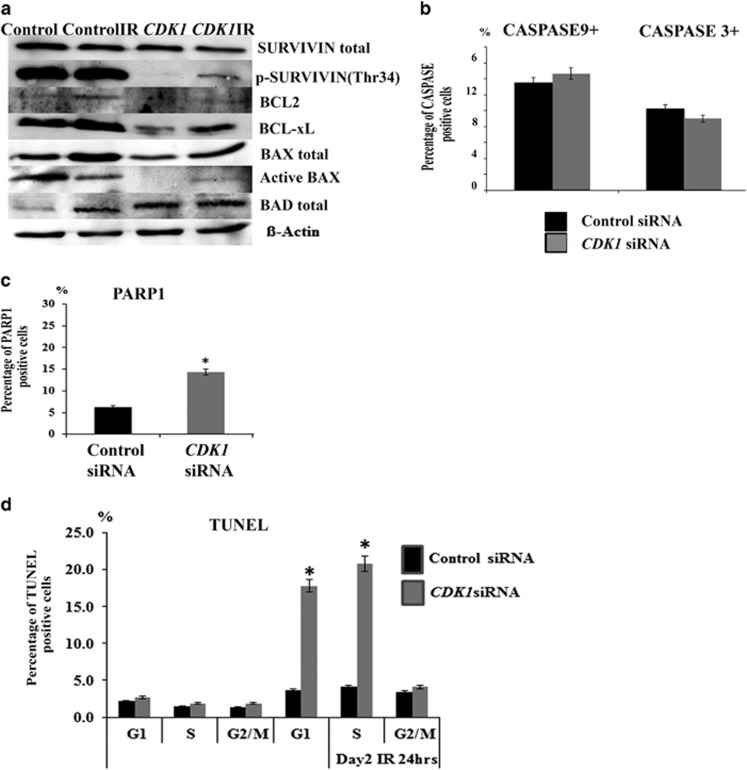
Knockdown of CDK1 in hESC results in PARP1 activation, but impaired apoptosis. (**a**) Western blot analysis showing downregulation of pro-survival proteins and activation of pro-apoptotic markers at 2 days post transfection of hESC with control and *CDK1* siRNAs in the absence and presence of IR (extracts were collected 16 h post IR). Images are representative of at least three independent experiments. *β-*Actin served as the loading control. (**b**) Graphical representation of flow cytometric analysis for caspase 9 and 3 activation in hESC at 2 days post transfection with control and *CDK1* siRNAs. Results are presented as mean±S.E.M. (*n*=3). (**c**) Graphical representation of flow cytometric analysis for PARP1 activation in hESC at 2 days post transfection with control and *CDK1* siRNAs. Results are presented as mean±S.E.M. (*n*=3). *T*-test analysis was carried out to assess the differences in gene expression between the control and CDK1 siRNA group. (**d**) Graphical representation of TUNEL analysis at each stage of cell cycle at 2 days post transfection with control and *CDK1* siRNA under normal culture conditions as well as IR. Results are presented as mean±S.E.M. (*n*=3), *t*-test analysis was carried out to assess differences in gene expression between the control and *CDK1* siRNA group, *P<0.05

The CDK1/CYCLIN B1 complex is able to interact and phosphorylate both pro- and anti-apoptotic proteins such as Bad, Caspase 9, Caspase 8, Caspase 2, Caspase3, Bcl-2, Bcl-xl, Mcl-1 and Survivin.^39, 40, 41, 42, 43^ We performed western blotting analysis and observed that the expression of anti-apoptotic/survival proteins (such as Survivin, BCL2, BCL-xL) was downregulated in the *CDK1* siRNA group (Figure 7a). In addition, the expression of pro-apoptotic protein BAD was increased upon *CDK1* downregulation. At the same time, the expression of active BAX (pro-apoptotic protein) was downregulated (Figure 7a); however, this is more likely to reflect the loss of pluripotency in hESC upon *CDK1* downregulation, as it has recently been shown that active BAX is only detected in undifferentiated hESC.^44^ We also performed flow cytometric analysis for expression of CASPASE 3 and 9, because it has been shown that the loss of phosphorylation on Thr34 of SURVIVIN can result in the disassociation of the Survivin-Caspase 9 complex and initiation of caspase 9-dependent apoptosis; however, we were unable to detect significant changes between the control and *CDK1* siRNA groups (Figure 7b). Another important apoptosis executor is PARP1 (poly ADP ribose polymerase 1), whose activity increases substantially in response to cellular stress.^45^ Flow cytometric analysis indicated significant upregulation of active PARP1 upon *CDK1* downregulation (Figure 7c); however, TUNEL analysis did not reveal significant differences in the number of apoptotic cells, suggesting an inability to commit to apoptosis in the absence of *CDK1* (Figure 7d). Similar data were obtained in hiPSCs (Supplementary Figure 5).

However, upon increased DNA damage (such as IR), commitment to apoptosis occurs (Figure 7d), suggesting that the threshold of accumulated DNA damage is the factor behind the hESC decision to expunge damaged cells either through induction of differentiation (steady state culture conditions) or apoptosis (IR conditions).

## Discussion

Our results suggest that downregulation of *CDK1* leads to the loss of hESC and hiPSC pluripotency and upregulation of a variety of lineage markers that are not only associated with differentiation to trophoectoderm but also extend to other lineages such as ectoderm, mesoderm and endoderm. That led us to speculate that the impacts of *CDK1* downregulation must go beyond a single interaction with OCT4.^17^ With this in mind, we screened the list of published target genes that are transcriptionally activated by pluripotency factors and also the range of substrates that are phosphorylated by CDK1 in hESC.^20, 46^ We found that *CDK1* is transcriptionally regulated by SOX2 and NANOG. ^20, 46^ Furthermore, a very recent paper that was published whilst this manuscript was under review showed that NANOG, a key pluripotency factor, is directly phosphorylated by CDK1.^47^ This later direct interaction has not been explored functionally; hence, it is impossible to conclude whether the impacts of CDK1 on maintenance of pluripotency are through direct interactions with pluripotency factors or indirectly through the large number of targets it may phosphorylate on human pluripotent stem cells.

Associated to these published data are also our important findings of polyploidy occurrence (14% of cell population) and multiple chromosomal abnormalities upon *CDK1* downregulation. For genomic instability to occur, there must be problems with the cell's ability to activate checkpoint signalling and repair DNA damage, its inability to activate and/or execute apoptosis and/or cell intrinsic problems during chromosome separation/cell division. To investigate which of these scenarios is prevalent when *CDK1* is downregulated, we first assessed how hESCs protect their genomes against DNA damage. We found that cells with reduced *CDK1* expression show almost three times as much accumulation of unrepaired DSBs compared with the control group, suggesting either a higher generation of DSBs or/and impaired ability to repair DSBs. Currently, it is unclear whether this is due to increased generation of DSBs during DNA replication or an inability to repair them *via* HR as CDK1 has been known to interact closely with the replication machinery as well as DSB DNA repair systems. Our data showing a very high percentage of cells with DSB foci in the S phase of the cell cycle are supportive of these two hypothesis; however, further work is needed to investigate this. Furthermore, upon exposure to IR, those cells are unable to maintain the G2/M arrest that is typical for hESC.^30^ Instead, a mixture of G1 and G2/M arrest was noticed for a prolonged time after administration of IR, leading us to investigate further the pattern of CHK1 and CHK2 activation upon *CDK1* downregulation. We noticed that whilst CHK1 activation and downstream p53-dependent transactivation was occurring, CHK2 activation was completely impaired. Although activation of CHK1 can compensate for some of the CHK2 functions, it is clear from our results that this is not sufficient to maintain a fully functional G2/M checkpoint arrest in response to DNA damage and to repair the DSBs arising under normal culture and stress conditions, when *CDK1* is downregulated in hESC.

Given the improper maintenance of G2/M checkpoint in the *CDK1* siRNA group, we went on to investigate mitotic progression using the mitosis-specific marker, phospho-Histone 3 (Ser10). We observed that a significantly higher number (15.7%) of cells in the *CDK1* siRNA group were at mitosis when compared with the control group (6%), suggesting an inappropriate escape from the G2 checkpoint back into the cell cycle, likely to result in tolerance to cells bearing DNA damage and/or unstable genomes. We analysed kinetochore, centrosome and centriole numbers and in all cases, we found a higher percentage of cells with altered distribution and number of these organelles. These organelles are essential for proper spindle microtubule formation, chromosome segregation and cell division; hence, the presence of multiple abnormalities of these organelles could lead to the formation of dicentric chromosomes as observed in the *CDK1* siRNA group. In addition to chromosomal abnormalities, *CDK1* downregulation also led to polyploidy in about 14% of the cell population corroborating previous data on somatic cells^48, 49, 50, 51^ and suggesting an important role for CDK1 in full and proper execution of mitosis and cytokinesis.

**Figure 8:**
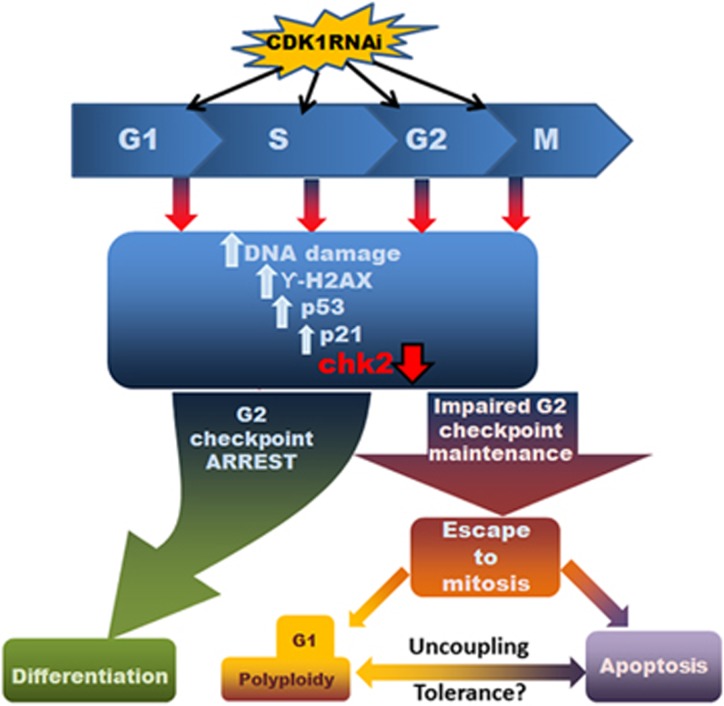
A schematic presentation of CDK1's function in maintenance of pluripotency and genomic stability in human pluripotent stem cells

In the absence of proper DNA damage repair and presence of mitotic deficiencies, execution of apoptosis for elimination of damaged cells with genomic instability becomes very important. Indeed, it has been suggested that hESCs prefer to eliminate damaged cells by apoptosis rather than undergo DNA repair.^52^ This was not the case as there was no significant increase in activation of CASPASE 3 and 9 in the *CDK1* downregulation group when compared with the control. We then investigated the activity of PARP-1 which is known to trigger the release of the mitochondrial apoptosis-inducing factor that promotes programmed cell death through a caspase-independent pathway.^53^ Although PARP1 activity was increased upon *CDK1* downregulation in hESC, we failed to detect a significant commitment to apoptosis in both hESC and hiPSC; however, administration of IR (which is known to cause increased DSBs) does result in enhanced apoptosis in hESC deficient for CDK1. Together, these data suggest that commitment to apoptosis in hESC and hiPSC is impaired in the absence of CDK1; however, this is dependent on the level of accumulated DNA damage, such that above a certain threshold, apoptosis is preferred rather than stem cell differentiation. Hence, loss of pluripotency and induction of differentiated state in the *CDK1* siRNA group could be the pluripotent stem cells response to restore the genomic stability by coupling the mitotic checkpoint control to execution of apoptosis upon induction of differentiation (refer to Figure 8 for a schematic summary).

## Materials and Methods

### Pluripotent stem cell culture and transfection experiments

The human H9 embryonic stem cell line (WiCell Research Institute, Madison, MI, USA) line was cultured on mitotically inactivated mouse embryonic fibroblasts and passaged as described by Neganova *et al.*^2^ Human iPSC (SB-NEO1) was generated from reprogramming of neonatal fibroblasts using the Sendai-based Cytotune 1 kit provided by Life Technologies (Paisley, UK). Full *in vitro* and *in vivo* characterisation was performed as part of the STEMBANCC European project and can be found in the StemBio Gateway. Human iPSCs were cultured the same way as hESCs on mitotically inactivated murine embryonic fibroblasts. A few passages prior to start of experiments, hESCs and hiPSCs were transferred to Matrigel-coated plates with feeder-conditioned media as previously described Neganova *et al.*^2^ Downregulation of *CDK1* was achieved using siRNAs: CDK1 siRNAs Validated Stealth RNAi DuoPak [CDC2VHS40172; duplex 1: (RNA) – CCU AGU ACU GCA AUU CGG GAA AUU U and duplex 2: (RNA) – GGA CAA UCA GAU UAA GAA GAU GUA G] from (Invitrogen Ltd, Paisley, UK; www.invitrogen.com) following the protocol reported in our previous publication.^2^ The cells were analysed at 24, 48, 72 and 96 h after transfection. Cell synchronisation at particular stages of the cell cycle was performed as described before.^2^

### Karyotype analysis

Karyotypes were determined by Standard G-Banding Procedure. At least 30 metaphases were analysed for each experiment.

### Western immunoblotting and immunoprecipitation

Protein extraction, western blotting and immunoprecipitation were performed as described in our previous publication.^2^ Primary antibodies used in this work were purchased from Santa Cruz Biotechnology Ltd (Middlesex, UK) and Cell Signalling (Danvers, MA, USA): Cdc2 p34; CDK2; SURVIVIN; p-SURVIVIN (Thr34); cyclin B1; p-Histone H3(Ser10); p-53; p-Chk1 (Ser345); p-Chk2 (Thr68); Chk1; Chk2; Cyclin A, p21, p27 and *β*-Actin. Bcl-xL; Bcl-2, BAX, active BAX; BAD; Cdc2; p-cdc2 (Tyr15); p-Cdc2 (Thr161); p-Cdc2 (Tyr15/Thr14); p-p53(Ser15) and p-p53(Ser20). The antibodies to *β*-actin and/or GAPDH (Abcam) were used after membrane stripping to confirm uniform protein loading. Antibody/antigen complexes were detected using ECL (Amersham Biosciences, Little Chalfont, UK; www.gelifesciences.com) and images were acquired using a luminescent image analyser FUJIFILM and LAS-3000 software (FUJI, Abingdon, UK; www.rndsystems.com).

### Quantitative reverse transcription-polymerase chain reaction

Total RNA was extracted using TRIzol reagent (Invitrogen) according to the manufacturer's instructions. Following DNase treatment using RQ1 DNaseI (Promega, Mannheim, Germany; http://www.promega.com), cDNA was synthesized using SuperScript Reverse Transcriptase (Invitrogen). qRT-PCR analysis was carried out using SYBR Green PCR master mix (Sigma Aldrich, Dorset, UK) and the primers are listed in supporting information Table 1. All samples were analyzed using an AB7900HT real-time analyzer (Life Technologies) and were normalized to *GAPDH*, *RPL13A* and *SDHA* expression.

### Cell cycle analysis

hESCs and hiPSCs were collected using Accutase (Chemicon, Temecula, CA, USA; www.millipore.com). Cell cycle analysis was performed using the CycleTest Plus DNA reagent kit (BD Biosciences, Oxford, UK) using a FACS Canto (BD Biosciences). The data were analysed using ModFit (Verity software House, Topsham, ME, USA; www.vsh.com) to generate percentages of cells in G1, S and G2/M phases. At least 10 000 cells were analysed in each experiment.

### Immunocytochemistry and confocal microscopy

Briefly, hESCs were cultured on Matrigel-covered glass slide flasks (SlideFlask, NUNC, Roskilde, Denmark; www.nuncbrand.co) in the presence of feeder-conditioned media. Cells were quickly washed with phosphate-buffered saline (PBS), prior to being fixed with 2% formaldehyde for 10 min and permeabilized with 0.1% Triton X-100 in PBS for 15 min at room temperature. Unspecific binding was blocked by incubation of samples in PBS containing 5% normal goat serum for 40 min. Staining with the mouse monoclonal anti-phospho-histone H2A.X (Ser 139; Millipore, Watford, UK; www.millipore.com) was carried out as described before.^2^ Slides were examined using a Zeiss confocal microscope (Carl Zeiss, Jena, Germany; http://www.zeiss.com). Quantification was performed by counting -H2A.X-positive foci in 150–200 nuclei per experiment.

For other immunocytochemical analyses, the cells were fixed in 4% (wt/vol) paraformaldehyde for 20 min. An additional permeabilization step in 0.2% (vol/vol) Triton X-100 in PBS was performed prior to staining with primary antibodies. Blocking step was performed by incubation in 1% (wt/vol) bovine serum albumin or alternatively in 10% (vol/vol) goat serum. Cells were incubated with primary antibodies overnight at 4 °C and secondary antibodies for 2 h. Primary antibodies used in this study are anti-Ki67, anti-Cdc2, anti-Cyclin B1, anti- Cyclin A and anti-p-Histone H3 (Ser10), all purchased from Santa Cruz Biotechnology. The nuclei were counterstained with DAPI. The bright-field and fluorescent images were obtained using a Zeiss microscope and the AxioVision software (Carl Zeiss). At least 100 cells were analysed for each technical replicate.

### TUNEL Assay

Analysis of DNA fragmentation was performed with APO-DIRECT kit (BD Pharmingen, Oxford, UK). Cells were prepared according to manual instructions and analysed by flow cytometry. Flow cytometric analysis (BD Biosciences LSRII) with two dyes, namely propidium iodide for staining total DNA and FITC-dUTP for staining the apoptotic cells, was carried out. At least 10 000 events were analysed in each experiment.

### CASPASE activity assay

APO LOGIX Carboxyfluorescein (FAM) Caspase Detection kit (Cell Technology, Kennesaw, GA, USA) was used to detect active caspases in living cells through the use of a carboxyfluorescein (FAM)-labelled peptide fluoromethyl ketone (FMK) caspase inhibitor (FAM-Peptide-FMK). The FAM-peptide inhibitor (FAM-LEHD-FMK) irreversibly binds to active caspase 9 and FAM-DEVD-FMK inhibitor to active caspase 3. Flow cytometry (LSRII, BD Biosciences) was used to measure the percentage of caspase 9-positive cells. At least 10 000 events were analysed in each experiment.

### Alkaline phosphatase staining

AP staining was carried out using the AP Detection kit according to manufacturer's instructions (Chemicon, Temecula, CA, USA). The bright-field images were obtained using a Zeiss microscope and AxioVision software (Carl Zeiss).

### Flow cytometric analysis for assessing apoptosis, DNA damage and cell proliferation

This was performed using a flow cytometric kit (cat. no. 562253; BD Biosciences) following the manufacturer's instructions. In brief, hESCs were labelled with BrdU, then fixed, permeabilized and treated with DNAse. Following this treatment, cells were simultaneously stained with PerCP-Cy5.5-labelled BrdU, PE-labelled anti-cleaved PARP and Alexa Fluor 647-labelled anti–*γ*H2AX. Cells were resuspended in staining buffer and analysed by flow cytometry. Cell cycle distribution was analysed by adding DAPI. At least 10 000 events were recorded for each sample. Annexin-V-PE apoptosis detection kit (BD Bioscience, Oxford, UK; www.bdbiosciences.com) was also used as described before in Neganova *et al.*^3^

### Statistical analysis

*T*-test analysis was used to assess differences between the control and *CDK1* siRNA group. The results were considered significant if *P*≤0.05.

### Indirect immunofluorescence for kinetochores, centrosomes and centrioles

For immunodetection of kinetochores, cells were fixed in 2% paraformaldehyde for 5 min at room temperature and in cold methanol for 5 min, then washed for 5 min in MBST buffer containing 10 mM 3-(N-Morpholino)- propane sulfonic acid (pH 7.2), 150 mM NaCl and 0.05% Tween- 20. The cells were blocked with 5% FBS in MBST buffer and incubated with human anti-centromere sera (CREST, 90C-CS-1058, Europa Bioproducts, Cambridge, UK; www.europa-bioproducts.com) diluted in MBST buffer containing 2% FBS. For immunodetection of pericentrin, hESCs were fixed in an 95% ethanol/1% acetic acid solution for 30 min on ice, transferred into PBS and permeabilized for 15 min with 0.1% Triton-X, blocked for 1 h with 1% bovine serum albumin in PBS (pH 7.4) containing 0.05% Tween-20 and incubated overnight at 4 °C with primary antibody against pericentrin (ab4448, Abcam, Cambridge, UK). For immunodetection of centrin, cells were fixed in ice-cold methanol:acetone (9 : 1) solution for 20 min, washed with PBS and permeabilized for 15 min with 0.1% Triton-X, blocked for 1 h with 1% bovine serum albumin in PBS (pH 7.4) containing 0.05% Tween-20 and incubated overnight at 4 °C with anti-centrin-2 (sc-27793-R, Santa Cruz Biotechnology). Incubations with secondary antibodies were carried out for 1 h at room temperature. Cell nuclei were counterstained with 4', 6-diamidino-2-phenylindole (DAPI, Sigma-Aldrich). Microscopy was performed using Zeiss microscope with Z-scanned (step 0.15 *μ*m) using × 63 objective and the AxioVision software (Carl Zeiss). At least 10 fields with were analysed for each of the three technical replicates.

## Figures and Tables

**Table 1:** The sequences of oligonucleotides used for qRT-PCR analysis.

*Gene Name*	*Primer (5'-3')*
*CDK1*	For: TTTTCAGAGCTTTGGGCACT Rev: CCATTTTGCCAGAAATTCGT
*OCT4*	For: AGCTCTGCAGAAAGAGTCCCAGG Rev: TGAGCCCCACATCGGCCTGT
*KLF4*	For: CCCAATTACCCATCCTTCCT Rev: CGTCCCAGTCACAGTGGTAA
*Lin28*	For: TCCTGCACTGTGTTCTCAGG Rev: AAAGCCAGCTCTTATTGGCA
*SOX2*	For: GGCAGCTACAGCATGATGCAGGACC Rev: CTGGTCATGGAGTTGTACTGCAGG
*NANOG*	For: TCCAGCTTGTCCCCAAAGCTTGC Rev: ACAGTCTCCGTGTGAGGCATCT
*CDX2*	For: GGCAGCCAAGTGAAAACCAG Rev: GGTGATGTAGCGACTGTAGTGAA
*GATA4*	For: ACACCCCAATCTCGATATGTTTG Rev: GTTGCACAGATAGTGACCCGT
*PAX6*	For: ACAGTCACAGCGGAGTGAATC Rev: ACTTTTGCATCTGCATGGGTC
*FGF5*	For: ATTTGCTGTGTCTCAGGGGAT Rev: CTGTGAACTTGGCACTTGCAT
*NESTIN*	For: CAGGAGAAACAGGGCCTACA Rev: TGGGAGCAAAGATCCAAGAC
*BRACHYURY*	For: TCAGCAAAGTCAAGCTCACCA Rev: CCCCAACTCTCACTATGTGGATT
*AFP*	For: CTTTGGGCTGCTCGCTATGA Rev: ATGGCTTGGAAAGTTCGGGTC
*GAPDH*	For: TGCACCACCAACTGCTTAGC Rev: GGCATGGACTGTGGTCATGAG
*RPL12A*	For: CCTGGAGGAGAAGAGGAAAGAGA Rev: TTGAGGACCTCTGTGTATTTGTCAA
*SDHA*	For: TGGGAACAAGAGGGCATCTG Rev: CCACCACTGCATCAAATTCATG

## Abbreviations

hESC: Human embryonic stem cells
hiPSC: induced pluripotent stem cells
DSBs: double strand breaks
siRNAs: small interfering RNAs
AP: alkaline phosphatase
IR: ionising irradiation
HR: homologous recombinationTUNEL
TUNEL: Terminal deoxynucleotidyl transferase dUTP nick end labeling
BrdU: 5-bromo-2-deoxyuridine
PE: Phycoerythrin
DAPI: 4',6-diamidino-2-phenylindole
