# Supplementary figures and images for: CDK1 plays an important role in the maintenance of pluripotency and genomic stability in human pluripotent stem cells
Source: Cell Death Dis. 2014 Nov 6;5(11):e1508–. doi: 10.1038/cddis.2014.464 (PMC4260724; doi:10.1038/cddis.2014.464)

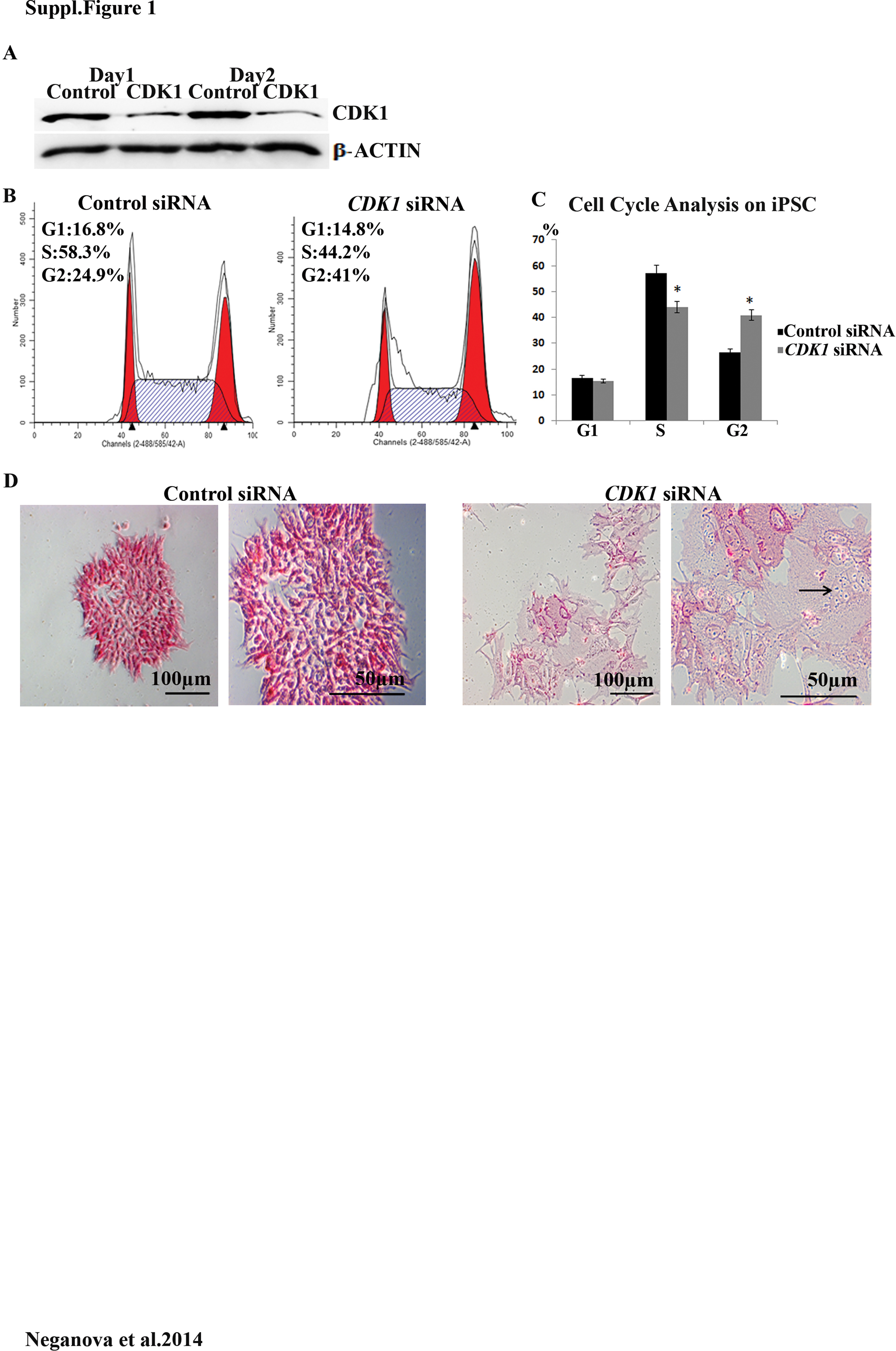

Supplement: Supplementary Figure 1 [file cddis2014464x1.tif]

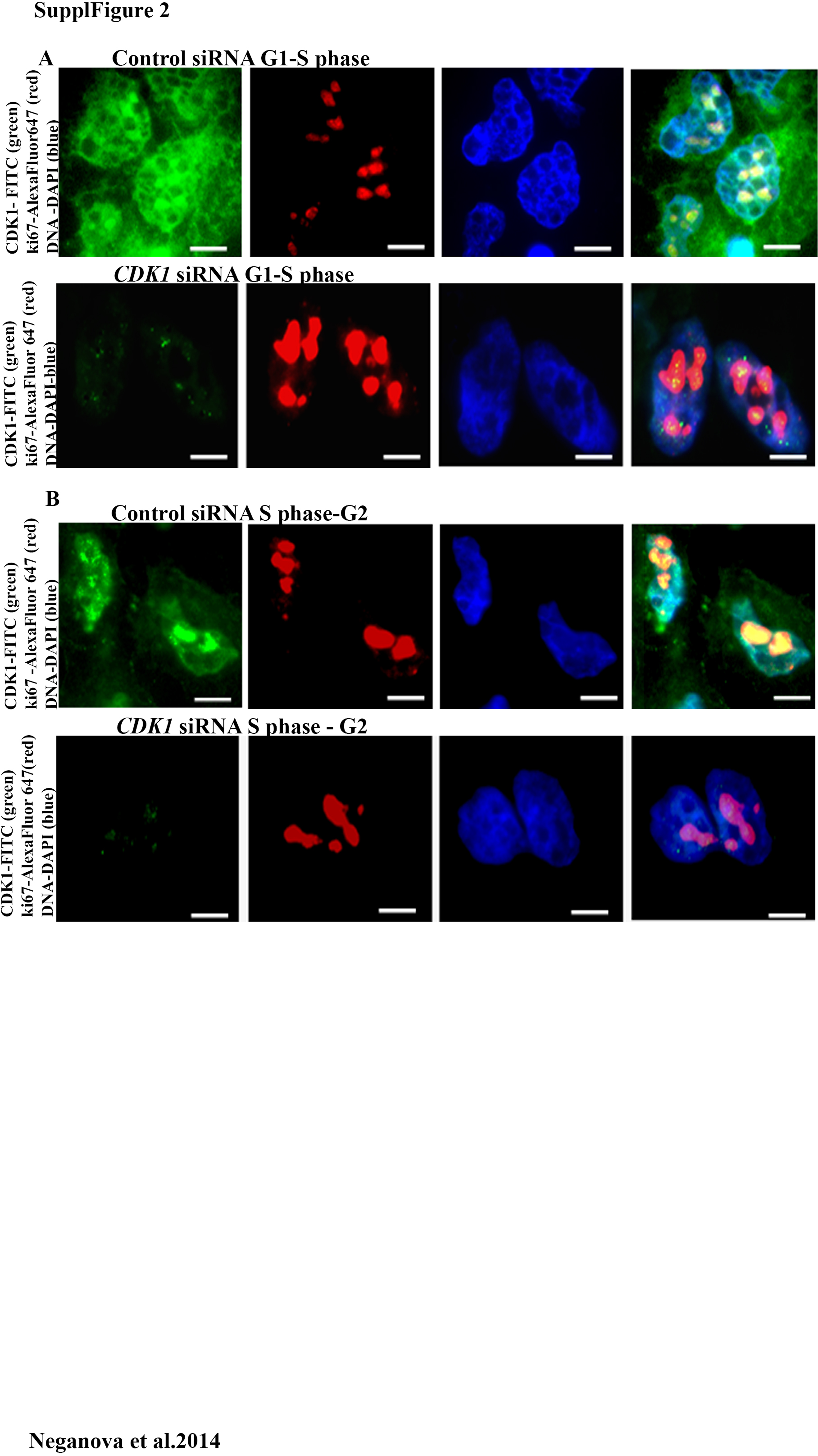

Supplement: Supplementary Figure 2 [file cddis2014464x2.tif]

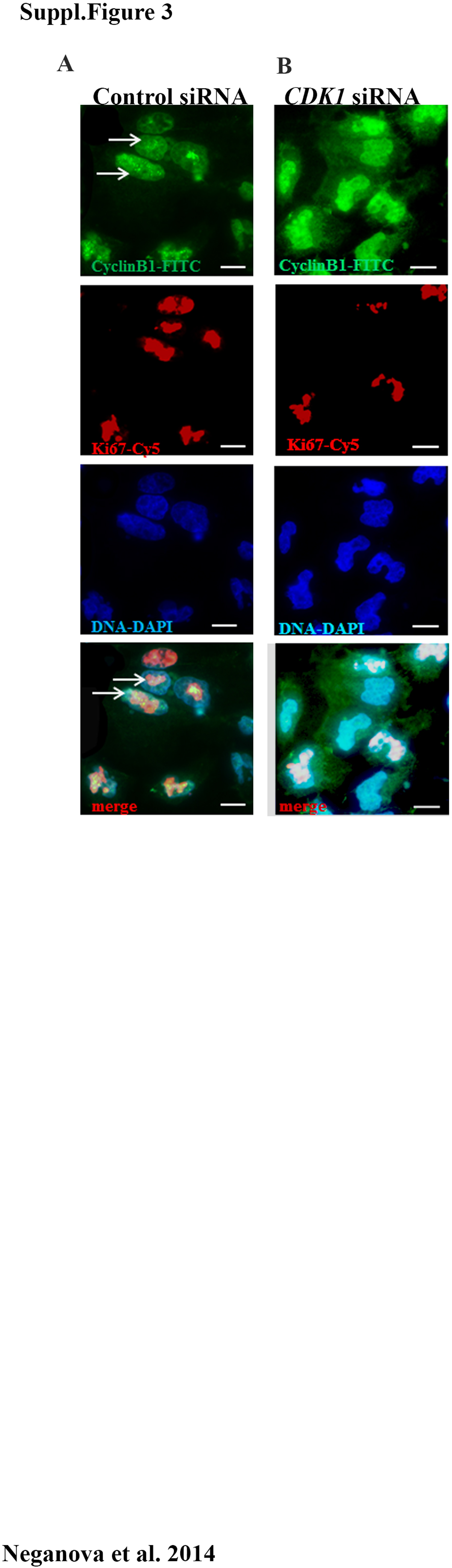

Supplement: Supplementary Figure 3 [file cddis2014464x3.tif]

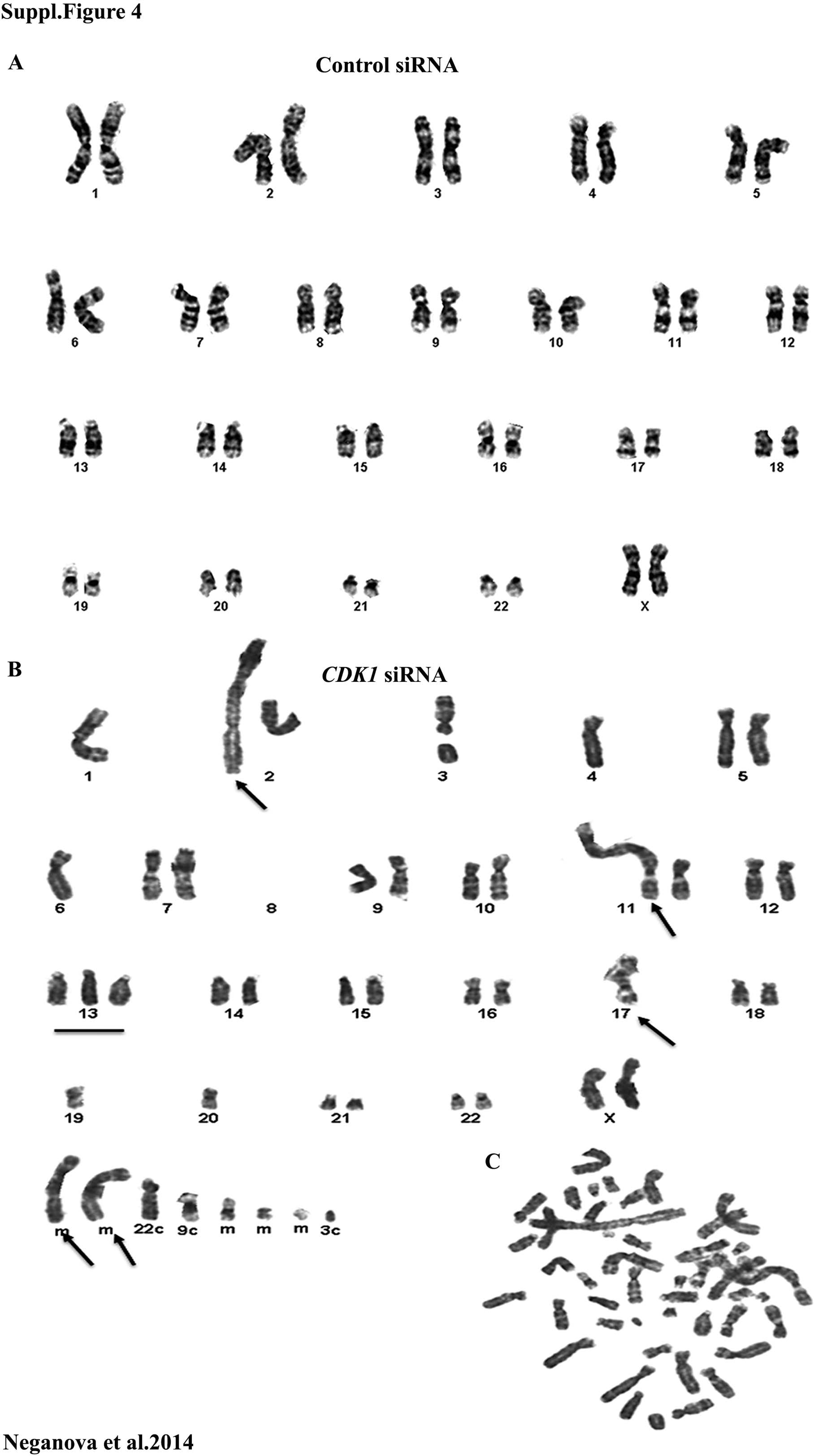

Supplement: Supplementary Figure 4 [file cddis2014464x4.tif]

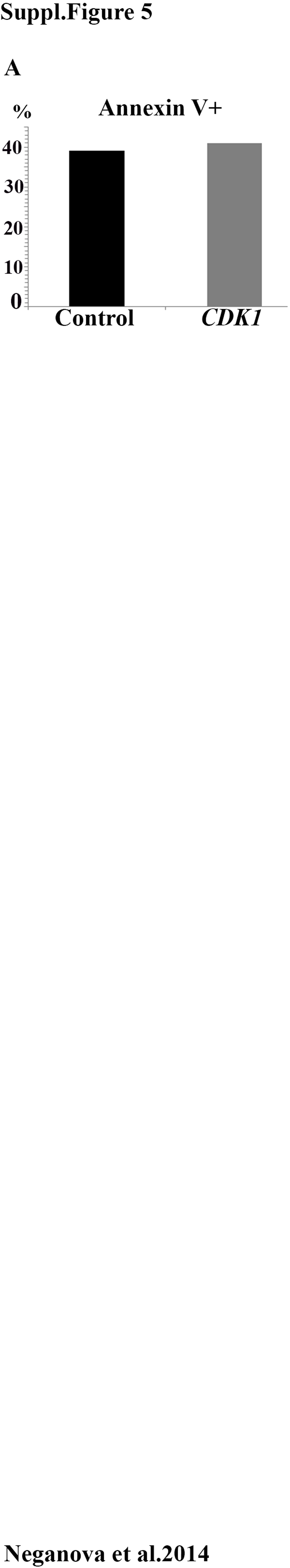

Supplement: Supplementary Figure 5 [file cddis2014464x5.tif]
