# Supplementary material for: CDK1 plays an important role in the maintenance of pluripotency and genomic stability in human pluripotent stem cells
Source: Cell Death Dis. 2014 Nov 6;5(11):e1508–. doi: 10.1038/cddis.2014.464 (PMC4260724; doi:10.1038/cddis.2014.464)
Supplement: Supplementary Information [file cddis2014464x6.doc]

**Supplementary Information**

**Suppl. Figure 1**. Down-regulation of *CDK1* in human iPSC. **(A)** Western blot analysis of CDK1 down-regulation in hiPSC. β – ACTIN was used as loading control. The data shown are representative of three independent Western blots; **(B)** Flow cytometric histograms showing cell cycle distribution in control and *CDK1* siRNA groups. The percentage of cells in each stage of the cell cycle is indicated in the top left corner of flow histograms. Data is representative of at least three independent experiments**; (C)** Graphic representation of the ModFit analysis of cell cycle distribution in the control and *CDK1* siRNA group, 48 hours post transfection with siRNAs. Results are presented as average ± SEM (n=3). *t*-test analysis was carried out to assess differences in gene expression between the control and *CDK1* siRNA group, * p < .05; **(D)** Alkaline-phosphatase positive staining was observed in hiPSC transfected with Control siRNA but differentiated morphology, lack of typical staining and polyploidy was observed in hiPSC transfected with *CDK1* siRNA at 48 hours post transfections. Images are representative of at least 3 independent experiments; black arrow points to a cell with numerous nuclei in the *CDK1* siRNA group.

**Suppl. Figure 2. (A)** Pattern of CDK1 distribution at G1–S stages revealed by immunofluorescence with anti CDK1 antibody (green), anti Ki67 (red) and DNA –DAPI (blue) in Control (upper row) and CDK1 siRNA group (lower row). (**B**) Prominent nuclear localisation of CDK1 (green) in cells treated with Control siRNA (upper panel) at late S-phase and G2 but absence of CDK1 positive staining in *CDK1*RNAi group (lower panel). Scale bar = 10 µm. Images are representative of at least three independent experiments.

**Suppl. Figure 3. Distribution of CyclinB1 is altered upon down-regulation of *CDK1*. (A)** hESC were transfected with Control siRNA and stained with anti-Cyclin B1 specific antibody (green) at 2 days post transfection. Note nuclear accumulation of Cyclin B1 during S-phase/G2 transition shown by white arrows and verified by Ki67 (red). DNA was counterstained with DAPI. The data shown are representative images of at least three independent experiments. Scale bar = 10µm; **(B**) Representative immunofluorescent images of cytoplasmic and nuclear distribution of Cyclin B1 (green) during S-phase/G2 transition (Ki67, red) at 2 days post transfection with *CDK1* siRNAs. Note cytoplasmic and nuclear expression of CYCLIN B1 during S-phase/G2 transition upon *CDK1* down-regulation. The data shown are representative images of at least three independent experiments. Scale bar = 10 µm

**Suppl. Figure 4. Down-regulation of *CDK1* induces numerous chromosome abnormalities in hESC (A)** Normal karyotype (46XX) of hESC transfected with control siRNA at 2 days post transfection; **(B)** Abnormal karyotype of hESC transfected with *CDK1* siRNAs at 2 days post transfection. Arrows indicated aberrant 5 dicentric chromosomes; **(C)** Metaphase plate of (**B**). These are representative images of at least three independent replicates.

**Suppl. Figure 5**. Graphical representation of Apoptosis analysis carried out in control and CDK1 siRNA cells at 48 post transfection. Results are presented as mean ± SEM (n= 3).
